# Acute Tramadol-Induced Cellular Tolerance and Dependence of Ventral Tegmental Area Dopaminergic Neurons: An In Vivo Electrophysiological Study

**DOI:** 10.32598/bcn.9.10.180

**Published:** 2019-05-01

**Authors:** Shabnam Khodayari, Firouz Ghaderi Pakdel, Parviz Shahabi, Somayyeh Naderi

**Affiliations:** 1. Neurophysiology Research Center, Urmia University of Medical Sciences, Urmia, Iran.; 2. Department of Physiology, School of Medicine, Urmia University of Medical Sciences, Urmia, Iran.; 3. Neuroscience Research Center, Tabriz University of Medical Sciences, Tabriz, Iran.; 4. Danesh Pey Hadi Co., Health Technology Incubator Center, Urmia University of Medical Sciences, Urmia, Iran.

**Keywords:** Tramadol, Dopamine, Ventral Tegmental Area, Firing rate, Tolerance, Dependence

## Abstract

**Introduction:**

Ventral Tegmental Area (VTA) is a core region of the brainstem that contributes to different vital bio-responses such as pain and addiction. The Dopaminergic (DA) cellular content of VTA has major roles in different functions. This study aims to evaluate the cellular effect of tramadol on the putative VTA-DA neurons.

**Methods:**

Wistar rats were assigned into three groups of control, sham, and tramadol-treated. The animals were anesthetized and their VTA-DA neuronal activity was obtained under controlled stereotaxic operation. The firing rate of the neurons was extracted according to principal component analysis by Igor Pro software and analyzed statistically considering P<0.05 as significant. Tramadol (20 mg/kg) was infused intraperitoneally.

**Results:**

Overall, 121 putative VTA-DA neurons were isolated from all groups. In tramadol-treated rats, the inhibition of the neuronal firing was proposed as tolerance and the excitation period as dependence or withdrawal. The Mean±SD inhibition time lasted up to 50.34±10.17 minutes and 31% of neurons stopped firing and silenced after 24±3 min on average but the remaining neurons lowered their firing up to 43% to 67% of their baseline firing. All neurons showed the excitation period, lasted about 56.12±15.30 min, and the firing of neurons increased from 176% to 244% of their baseline or pre-injection period.

**Conclusion:**

The tolerance and dependence effects of tramadol are related to the changes in the neuronal firing rate at the putative VTA-DA neurons. The acute injection of tramadol can initiate neuroadaptation on the opioid and non-opioid neurotransmission to mediate these effects.

## Highlights

- We studied the cellular effects of tramadol on the Ventral Tegmental Area (VTA), as a major part in the brain involved in addiction phenomenon.
- The dopaminergic VTA neuronal firings were extracted and assigned as tolerance and dependence to tramadol by showing the inhibition and excitation, respectively.
- Tramadol can induce tolerance and following dependence in acute administration.
- Although a single acute dose of tramadol cannot elicit the tolerance and dependence, on the cellular level, it could kindle the neurons to the progress of the addiction behaviors.

## Plain Language Summary

Drug addiction is a major maladaption of the brain in human populations. It could even start by prescription of drugs. The Ventral Tegmental Area (VTA) Dopaminergic (DA) neurons are the core brain region affected in addiction. Tramadol is introduced as a safe painkiller to replace morphine, but its dependence effects have not studied well. This study showed that a single dose of tramadol could produce the cellular signs of addiction by inducing tolerance and dependence in some VTA-DA neurons. Inhibition and excitation of the VTA-DA neurons showed that tramadol could kindle the neurons to progress toward maladaptions. These findings suggest that caution should be taken when using tramadol as an analgesic.

## 1. Introduction

Many researchers in the field of pain management are exploring different perspectives of analgesics and their side effects to provide and market safer analgesics. Furthermore, pain modulation is a highly focused research topic with respect to drug design and development. Analgesic agents have changed rapidly to have lower side effects and addiction, and improve analgesia, especially in chronic pain management (Yaksh, 2002). Although people complaining of chronic pain is increasing worldwide, analgesic drugs for their treatment are reportedly inadequate due to their side effects that are often intractable and sometimes irreversible.

Neuroplasticity of nociception in the brain depicts the rationale for pharmacological research therapy of pain (Fornasari, 2014). The opiate-based compounds have been very common in the field of analgesia for several decades, so the drugs that affect µ-opioid receptors (MOR) are considered as major analgesics. Synthetic µ-opioid agonists and or antagonists of MORs have been formulated for pain control widely (Pasternak, 2010; Trescot, Datta, Lee, & Hansen, 2008).

Tramadol (Ultram) has marketed recently for the pharmacotherapy of chronic pain such as neuropathic pain. It is commonly used as a co-medication or alone for pain alleviating with greater safety. Its public distribution due to lower susceptibility to addiction, make it one of the most chosen analgesics in many pharmacopeias (Grond & Sablotzki, 2004). Tramadol has fewer side effects and dependence than equianalgesic doses of strong opioids like morphine (Flor, Yazbek, Ida, & Fantoni, 2013; Moron Merchante et al., 2013; Pergolizzi, Taylor, & Raffa, 2011; Rosenberg, 2009; Savoia, Loreto, & Scibelli, 2000).

Tramadol has an affinity to the Morphine Opioid Receptors (MORs) but this affinity is 6000 times less than that of morphine (Raffa et al., 2012). Tramadol and opioids are used for third-line treatment of neuropathic pain, to lower the outcomes of the first- and second-line agents and obtain the safety in long-term administration (Amescua-Garcia et al., 2017; O’Connor & Dworkin, 2009). Besides its action on MORs, tramadol has anti-depressive effects through reuptake inhibition of serotonin and noradrenaline (Barber, 2011; Bloms-Funke, Dremencov, Cremers, & Tzschentke, 2011; Caspani, Reitz, Ceci, Kremer, & Treede, 2014). It may be more effective than methadone in controlling opioid withdrawal symptoms and is considered as a potential replacement for methadone therapy (Zarghami, Masoum, & Shiran, 2012).

In addition to the safety of tramadol compared to morphine, there are some side-effects in case of therapeutic or overdose administration. There are post-subacute and chronic tramadol toxicities, also seizure is clinically a profound side effect that may occur. The social-based prevalence of tramadol-induced seizure is 8% to 14% but the hospitalized data are reported as 15% to 55% with mostly one episode (Emamhadi, Sanaei-Zadeh, Nikniya, Zamani, & Dart, 2012; Matthiesen, Wohrmann, Coogan, & Uragg, 1998; Spiller et al., 1997). The tramadol-induced seizure is not prevented by naloxone, a µ-opioid receptor antagonist (Eizadi-Mood, Ozcan, Sabzghabaee, Mirmoghtadaee, & Hedaiaty, 2014). However, with serotonin depletion in the rats, the tramadol-induced seizure is enhanced (Fujimoto et al., 2015). These reports show a strong link between tramadol and brain monoamines.

The mechanism(s) of analgesic effects of tramadol has remained controversial; the contribution of µ-opioid receptors (MORs) along with non-MOR elements has been evaluated (Minami, Sudo, Miyano, Murphy, & Uezono, 2015). Tramadol may change ion channels conductance by G-Protein Coupled Receptors (GPCRs) which can affect monoamine transporters (Minami, Ogata, & Uezono, 2015). Tramadol has rewarding effects (mediated by MOR activation) on rats with sciatic neuropathic pain. The dopamine level in the rats’ nucleus accumbens is significantly increased by either tramadol or its metabolite (Nakamura et al., 2008). In human cases, there are some reports on the rewarding and withdrawal effects of tramadol (Reeves & Cox, 2008), but its use for depression treatment is limited due to reported side effects (Ansermot, Chocron, Herrera, & Eap, 2015).

The overlap of chronic pain and depression has been reported by some epidemiologic studies (Bair, Robinson, Katon, & Kroenke, 2003) in which the cortical and subcortical structures are involved in the perception of different aspects of pain (Bushnell, Ceko, & Low, 2013). The mesolimbic Dopaminergic neurons have a modulatory effect on pain perception. There is evidence of dysfunction of the nucleus accumbens and Ventral Tegmental Area Dopaminergic (VTA-DA) neurons to initiate excessive pain in the animal model (Saade, Atweh, Bahuth, & Jabbur, 1997).

The VTA contains dopamine, Gamma-Aminobutyric Acid (GABA), glutamate-releasing neurons, and a few other types of neurons. The major population of its neurons (about 65%) is Dopaminergic, and about 30% GABAergic (Holly & Miczek, 2016; Nair-Roberts et al., 2008; Yamaguchi, Wang, Li, Ng, & Morales, 2011). The VTA-DA neurons as A10 group project to nucleus accumbens, amygdala, hippocampus, prefrontal cortex, and limbic system (Albanese & Minciacchi, 1983; Fallon, 1988) to create two major projecting systems: mesolimbic and mesocortical systems.

The mesolimbic system regulates motivational processes but the mesocortical system is related to cognitive and motor functions (Bjorklund & Dunnett, 2007; Le Moal & Simon, 1991; Merrill, Friend, Newton, Hopkins, & Edwards, 2015; O’Connell & Hofmann, 2011; Pierce & Kumaresan, 2006). The VTA-DA neurons is a key neuronal substrate of the limbic system for explaining reward, motivation, addiction, and neuropsychiatric illness (Luscher & Malenka, 2011; Nestler & Carlezon, 2006; Wise, 2004). The VTA-DA neurons have µ-opioid receptors that are involved in the excitation of dopamine in the nucleus accumbens (Johnson & North, 1992).

Although the rewarding effects of the tramadol have been documented, the precise mechanism of its action is not well known. Tramadol could mimic the opioid effects but it seems that its side effects are related to its opioid and non-opioid effects. This study was designed to evaluate the cellular effect of the acute intraperitoneal application of the tramadol on the firing rate of VTA-DA neurons with extracellular single neuron recording techniques. The units with sable spontaneous tonic activity without pattern changes were isolated and studied in this Research.

## 2. Methods

### 2.1. Ethical approval

Urmia Medical Science Research Ethics Committee (UMSREC) reviewed all procedures and experiments as the local referral Biomedical Committee for Research Ethics. Protocols and guidelines were carried on in accordance with the National Institutes of Health (NIH) for the care and use of experimental animals. The Animal Laboratory Center of Urmia University of Medical Sciences precisely outlined these protocols and guidelines. Supervision of animal protocols was carried out according to the research design.

### 2.2. Animals

Healthy male Wistar rats (Purchased from Pasteur Institute, Tehran, Iran, weight 180–220 g) were housed (three per cage) at 12:12 h light/dark cycle (7:00 AM–7:00 PM, light on at 7:00 AM) and controlled temperature (22±2°C) with the food and water ad libitum. The animals were divided into 3 groups of control, sham, and tramadol-treated (20 mg/kg, single dose, IP). The firing of putative VTA-DA neurons was recorded under urethane anesthesia. A single dose of urethane (1.2 g/kg) was usually sufficient for the entire recording, but the booster doses (15% to 25% of the initial dose) was available in case of observing any discomfort sign. Animal body temperature was continuously monitored to be maintained at ∼37°C during the experiment. Finally, the stereotaxic coordinates for bregma and the interaural line were calculated from the Bregma Zero-Zero (BZZ) plane and a burr hole for electrode placement was drilled to access the VTA according to the Paxinos and Watson rat brain atlas (Paxinos & Watson, 2007).

### 2.3. In vivo extracellular electrophysiological recording of VTA-DA neurons

Extracellular multi-unit electrophysiological activity of the putative VTA-DA neurons was recorded under urethane anesthesia as described previously. Briefly, after anesthesia the rat was mounted into the stereotaxic frame (Steolting, USA), its skull was exposed, and the stereotaxic landmarks were determined for opening a burr hole on the BZZ plane. Glass microelectrodes (in vitro impedance 3–6 MΩ, at 1 kHz) were filled with pontamine sky blue (2.0%) in sodium acetate solution (0.5 M) and inserted into the VTA (bregma=−6.84, ML=±0.5 and DV=8.6 mm at the BZZ plane in adjusted coordinates, angle=15° coronally).

The signals were amplified (10000×) and filtered (300–3000 bandpass, sample rate=50000) for obtaining through a high speed, isolated USB port on a PC running Windows 7.0 Premium Pro. Only spontaneous active right putative VTA-DA neurons were recorded and analyzed. The criteria explained in previous articles were applied in the analysis software protocol. The main criteria were low discharge rate (<10 spikes/second), long-duration of spike (2.5 ms) and a triphasic (+/−/+) or biphasic spike commonly with a notch in the positive component (Hyland, Reynolds, Hay, Perk, & Miller, 2002; Nimitvilai, Arora, McElvain, & Brodie, 2012).

Only units with stable firing rates were recorded while non-stable ones were ignored. The first 10-min was recorded as baseline spiking after stability. In the sham group, the drug vehicle (fresh normal saline) was infused intraperitoneally then the records were continued to investigate the confounding effects. In the control group, the firing of putative VTA-DA neurons was recorded up to 120 minutes to evaluate recording condition and stability. The units were isolated with Igor Pro 6.0 (Wavemetrics Inc. USA) and Principal Component Analysis (PCA). Peri-Stimulus Time Histogram (PSTH) was calculated off-line for all recordings and the average firing rate in the pre- and post-infusion periods were used for each recording in statistical analysis.

### 2.4. Data analysis

The Kolmogorov-Smirnov (K-S) test was used as a goodness of fit-test for the statistical probability distribution of data. The 1-way repeated measures ANOVA was used for comparing intra- and inter-groups mean firing rates with P<0.05 setting as the significance level. The information is presented as Mean±SEM and data analysis was done in Igor Pro 6.0 software.

### 2.5. Histological verification

At the end of the experiments, the recording site was marked by passing −20 µA electrical current through the recording electrode for 10 minutes to deposit pontamine sky blue dye. The rats were deeply anesthetized, perfused transcardially with phosphate-buffered (10%) formalin solution (4%) and their brains were removed and fixed in perfused solution. Then, 40-µm coronal sections were cut with a microtome (SLEE, London) to explore the electrode tip location and the trajectory path under a light microscope.

### 2.6. Drugs and materials

Drugs and chemicals used were as follows: tramadol, formalin, pontamine sky blue, fast cresyl violet, urethane (Sigma-Aldrich, USA), sodium acetate and sodium chloride (Merck, Darmstadt, Germany), polyethylene microtube (A–M system, USA), and Hamilton micro-syringes (Hamilton Bonaduz AG, Switzerland).

## 3. Results

### 3.1. Summary

Briefly, a single dose of the tramadol was infused intraperitoneally and the acute tramadol effect on the putative VTA-DA neurons was studied. Tramadol infusion inhibited the neuronal firing of putative VTA-DA neurons (acute neuronal tolerance) in a short time but the firing of neurons returned to baseline for a while and then increased up to double the rate of baseline (acute neuronal dependence or withdrawal). The average firing rate of the putative VTA-DA neurons can explain some of the side effects of acute tramadol administration.

### 3.2. The putative VTA-DA neuronal spike signature

**Figure 1.**
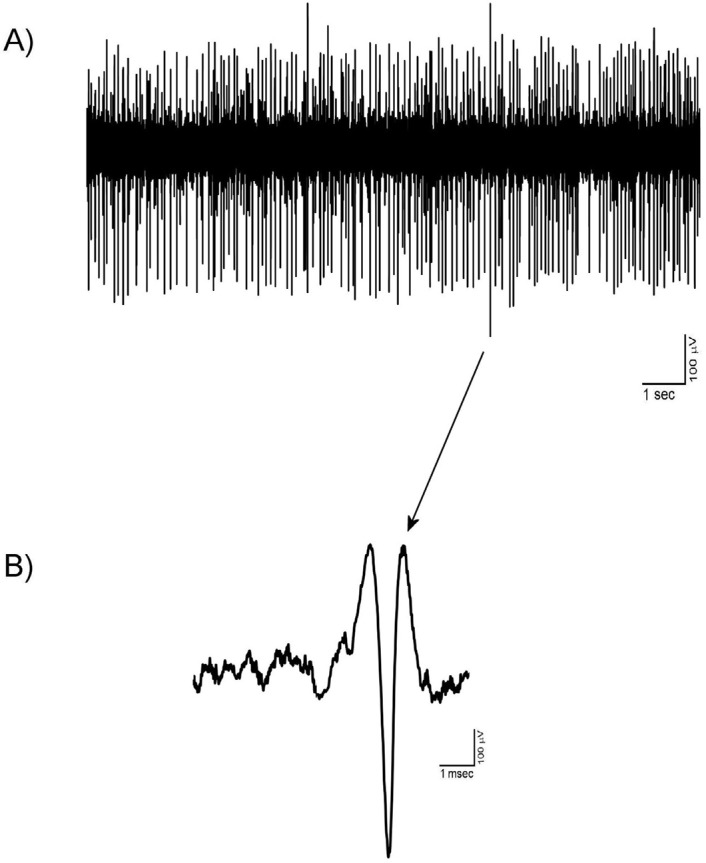
A typical multiunit recording of the VTA Dopaminergic neurons in the control group A. A trace of the multiunit recording; B. A typical spike signature of VTA Dopaminergic neuron isolated from the recording. The VTA Dopaminergic neurons have commonly a three-phase extracellular spike shape with the firing rate less 10 spikes/second. The showed neuron had triphasic (+/−/+) shape with a notch at the beginning of the first + phase. The mean amplitude of the showed neuron was about 800 µV peak-to-peak. VTA Dopaminergic neurons showed a low spontaneous firing rate (<10 spikes/second).

The putative VTA-DA neurons with stable and tonic spiking isolated in Vall recordings. Totally, 125 VTA-DA neurons were obtained from all groups and recorded for up to about 3 hours. The spike shapes of the extracted putative VTA-DA neurons has commonly a triphasic model. In Figure 1, a typical spike signature is shown. The Mean±SD spike amplitude in the recordings was 520±87.5 µV peak-to-peak. Putative VTA-DA neurons also showed a low spontaneous firing rate (<10 spikes/second). As shown in Figure 1, the main spike signature is the low firing rate with long-duration.

### 3.3. The histogram of the putative VTA-DA neurons in control and sham groups

**Figure 2.**
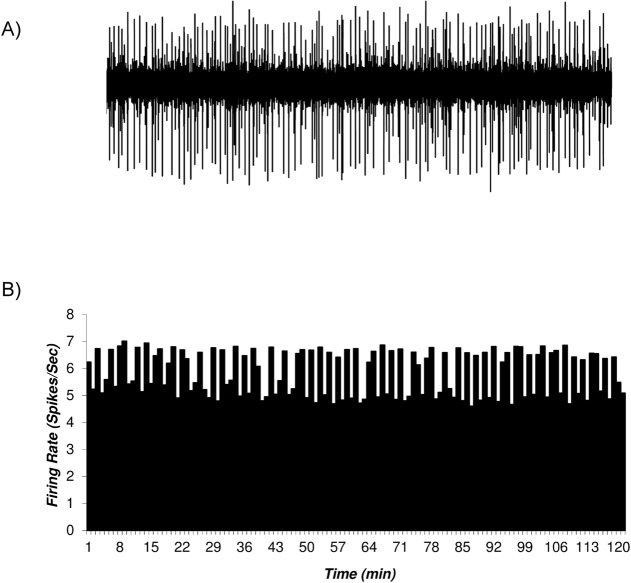
A sample of the multiunit firing of the putative VTA Dopaminergic neurons in the control group A. A sample trace of the multiunit firing of the VTA Dopaminergic neurons in the control group; B. The histogram of the firing of the VTA Dopaminergic neurons of the control group. In the control group (naïve) animals, the VTA Dopaminergic neuronal firing under standard condition was recorded up to 120 min. The Mean±SD firing rate of the neurons in the control group was 4.4±0.61 spikes/second. The mean firing in each minute was calculated from the averaged firing in each second in every minute. The data are presented as Mean±SD.

**Figure 3.**
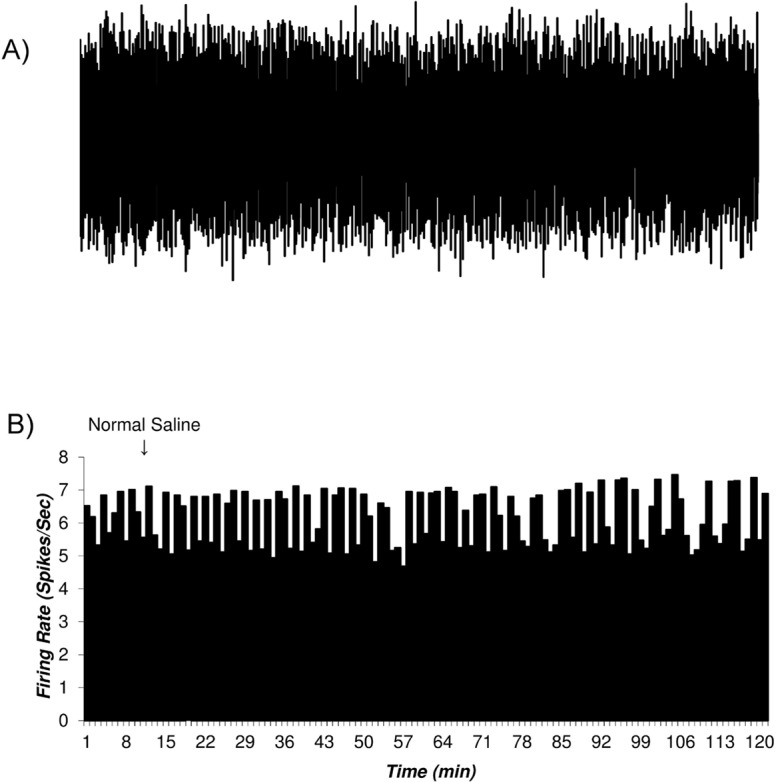
The firing of the putative VTA Dopaminergic neurons in the sham group The tramadol vehicle (sterile normal saline) applied intraperitoneally, showing pre- and post-injection firing rate of the selected neurons evaluated. A. The firing of a sample of VTA Dopaminergic multiunit neurons in the sham group. In the sham groups, the putative VTA Dopaminergic neuronal firings were recorded under standard condition up to 120 min. The injection of the tramadol vehicle (sterile normal saline, in the 10th min) done by 30-gauge syringe; B. The histogram of the neuronal firing rate of the recorded neurons. Vehicle had no significant effect on the neuronal firing. The paired Student t-test was used for statistical analysis.

The firing rates of putative VTA-DA neurons in the rats of the control (naïve) and sham groups are shown in Figures 2 and 3. Figure 2 shows the firing of a sample putative VTA-DA neuron in the control group. The Mean±SD firing rate of this neuron was 4.4±0.61 spikes/second during 120 min. The mean firing in each minute was calculated from the averaged firing in each second in every minute. Figure 2 shows a stable firing without any pattern change. Figure 3 shows the firing of a sample of putative VTA-DA neuron in the sham group, in the pre- and post-vehicle injection (normal sterile saline as a vehicle of tramadol), in the 10^th^ minute of recording. The Mean±SD sample neuronal firing rate was 6.13±0.82 spikes/second. Saline had no significant effect on the neuronal firing rate.

### 3.4. The histogram of the putative VTA-DA neurons in tramadol-treated group

**Figure 4.**
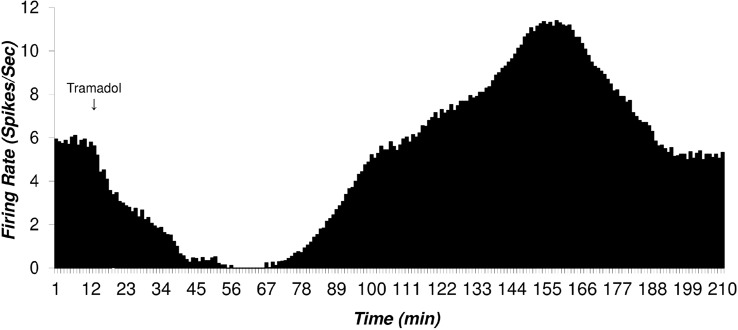
The histogram of the mean firing of a typical putative Ventral Tegmental Area Dopaminergic (VTA-DA) neuron after infusion of tramadol (20 mg/kg, single dose, IP, in the 10^th^ minute) The pre- and post-injection neuronal firing were recorded until the last firing returned to the baseline of pre-injection one. The typical decrease or inhibition of firing and consecutive excitation were seen in the Figure. The recorded neuronal firing was obtained and averaged in 1 second in each minute with 1 ms bin size for the evaluation of pattern change. The pre-stimulus time histogram was compared between the pre- and post-injection to evaluate statistical significance. The recording of neuronal firing continued until the firing of post-injection period returned to the pre-injection period rate. In all recordings of tramadol treatment, neuronal firings showed two distinct phases in the firing rate. A short time after tramadol injection, the neuronal firing decreased. This period is called the inhibition period or neuronal tolerance. In the presented sample, the neuron was silent about 13 min and in the inhibition period, the Mean±SD of firing was 1.83±1.94 spikes/second. In this neuron, the duration of inhibition was 93 min. The second phase began with an increase in the firing rate as excitation or dependence period. The mean firing rate of the excitation period was 8.33±1.87 spikes/second and the maximum rate was 11.4 spikes/second. The duration of the excitation period was 87 min. The final Mean±SD baseline firing was 5.2±0.16 spikes/second. The firing rate of the baseline, inhibition, and excitation periods were significantly different (using 1-way analysis of variance, Tukey’s post-hoc test, P<0.001).

The acute effect of the Intraperitoneal (IP) application of tramadol (20 mg/kg, single dose) on putative VTA-DA neurons firing was evaluated. Figure 4 shows a typical firing of a putative VTA-DA neuron to the application of tramadol. The Mean±SD baseline pre-injection firing rate of the neuron was 5.84±0.17 spikes/second. Tramadol (20 mg/kg, IP) was injected in the 10th minute of recording. The recording of neuronal firing continued until the firing of post-injection period returned to the pre-injection period rate.

In all selected recordings of tramadol-treated animals, neuronal firings showed three distinct phases. A short time after tramadol injection, the neuronal firing decreased. This period called inhibition period or the tolerance to tramadol. In the showed sample, the neuron was silent about 13 min and in the inhibition period, the Mean±SD firing rate was 1.83±1.94 spikes/second. The inhibition period was calculated from the minute that the mean firing rate decreased significantly from the previous minute. The termination of the inhibition period was calculated when the firing rate returned to the pre-injection one. In the sampled neuron, the duration of inhibition was 103 min.

The inhibition of neurons was followed by an increase in the firing rate. This period was called excitation period or withdrawal sign (dependence period). The duration of the excitation phase was calculated by the statistically significant difference of the post-inhibition firing the minute that firing rate returned to the pre-injection period and then increased. The Mean±SD firing rate of the excitation period of the showed sample in Figure 4 was 8.33±1.87 spikes/second and maximum rate was 11.4 spikes/second. The final baseline firing was calculated and continued for about 15 min. The duration of the excitation period was 87 min. The final Mean±SD baseline firing rate was 5.2±0.16 spikes/second.

**Figure 5.**
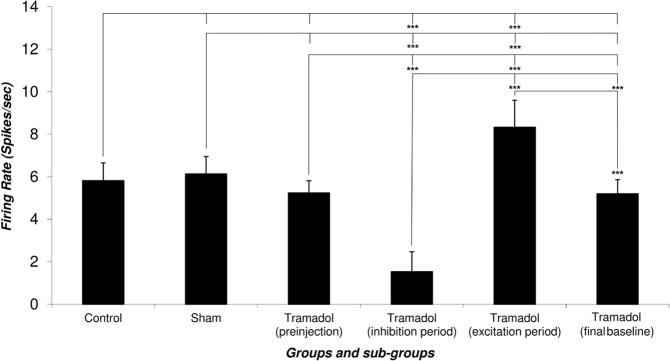
The bar chart of the firing rates of the putative Ventral Tegmental Area Dopaminergic (VTA-DA) neurons The chart shows the comparison of the averaged neuronal firing rate of the control and sham groups with the different periods of responses to tramadol injection (pre-injection, post-inhibition, post-excitation, and final baseline). Tramadol was injected in the 10th minute and the neuronal firings were obtained until the return of pre-injection firing rate. The mean firing rate of pre- and post-injection calculated for determination of the statistical difference. The Figure shows a significant statistical difference between pre- and post-injection of tramadol. The two distinct phases of tramadol effects (inhibition as tolerance and excitation as dependence) are seen in the firing rates of the neurons (using 1-way ANOVA, Tukey’s post hoc test, ^***^ P<0.001).

Figure 5 depicts the Mean±SD firing rate of the control group neurons (n=33) during 120 minutes of recording. The Mean±SD firing of control groups was 5.82±0.83 spikes/second. The Mean±SD firing rate of the sham group neurons (n=31) was 6.13±0.82 spikes/second and did not change until 120 min of recording. The Mean±SD neuronal firing rates of the tramadol-treated group were 5.24±0.57, 1.53±0.94, 8.33±1.27, 5.2±0.66 spikes/second in the pre-injection, inhibition period, excitation period, and final baseline, respectively.

Statistical comparison of the control and sham group’s neuronal firing rate showed no significant effect of the vehicle in putative VTA-DA neuronal firing and pre-injection and final period in the tramadol-treated groups. The neuronal firing rate decreased in the inhibition period (cellular tolerance) and increased in the excitation period (cellular withdrawal or dependence) in comparison with pre-injection and final baseline firing of tramadol-treated, control, and sham groups. On the whole, 57 putative VTA-DA neurons were isolated in the tramadol-treated group. Overall, tramadol-induced inhibition of neurons began 6±0.5 minutes after the injection and lasted up to 50.34±20.17 min on average. Thirty-one percent of neurons stopped firing and silenced after 24±3 min on average but the remaining neuron lowered their firing down to 43% to 67% of their baseline firing.

The inhibited neurons showed a withdrawal response to tramadol with increasing their firing. In the excitation period, the neurons showed excitation that lasted about 56.12±15.30 min on average. In this period, the neurons increased their firing from 176% up to 244% of their baseline or pre-injection period.

## 4. Discussion

In this study, the results showed that the acute effect of tramadol on some putative VTA-DA neurons has two consecutive inhibitory and excitatory effects. We refer the inhibitory effect as cellular tolerance and excitatory response as cellular withdrawal or dependence. The inhibitory response lasted about 50±20 min and the excitatory period lasted about 56±15 min on average. The biphasic response of the recorded neurons showed that some of the Dopaminergic neurons of the VTA might be involved in the neuronal adaptation to acute effects of tramadol. This study is the first report about the response of the putative VTA-DA neurons to tramadol at the extracellular electrophysiological levels. The indirect evidence can explain the basis of the responses but further pharmacological evidence is needed to elucidate the tramadol effects on these neurons.

### 4.1. Tolerance and dependence: neuroadaptation of the reward system

The Substance Use Disorder (SUD) is a neurochemical dysregulation of the brain reinforcement system. After long-term drug use, brain structural and functional abnormalities create different somatic and psychologic outcomes (Russo et al., 2010). The abused drugs associated with SUDs could elevate dopamine to supraphysiological levels and subsequent pre- or post-synaptic events occur based on receptor post-signaling cascade. The genomic and non-genomic effects are related to the type and duration of drug use. The involvement of cyclic Adenosine Monophosphate (cAMP) via G-proteins are the key focal point to the events (Nestler, 2012).

The contribution of VTA in the reward circuitry is proved by its roles in the conditional and or non-conditional aspects of SUDs (Volkow et al., 2010). The potency of drugs to activate or inhibit the neuronal elements of the reward circuitry can produce positive subjective effects to use a given substance. Drug craving originated from continued desire will produce the tolerance and withdrawal signs that may end in physical and psychological problems. After long-term drug consumption, the synaptic plasticity can produce maladaptation, which is responsible for tolerance and withdrawal.

The intensity of these SUDs symptoms varies greatly across different classes of substances but undoubtedly contributes to its continuous use. The treatment of withdrawal-induced alterations calls for an understanding of the biochemistry and neuroadaptations to chronic or acute use (Everitt, 2014). Drug-induced neuroadaptations after repeated use initiate two clinical consequences; drug tolerance and withdrawal. The need to achieve proper effects with incremental doses expresses the tolerance and withdrawal is a syndrome that occurs at the disruption of the drug. The neuronal basis of tolerance and withdrawal overlaps and withdrawal appears in the tolerated patients.

In tolerance mode, the responsiveness of the neuronal receptors to stimulation by drugs gradually decreases and the greater amounts of drug should be used to produce the desired effect. The cellular mechanism for tolerance is related to the neurotransmitter receptors, cAMP, Protein Kinase A (PKA), and cAMP Response Element-Binding (CREB) signaling molecules. The genomic alteration could prolong and enhance the tolerated responses in parallel with neurotrophic factors (Trujillo, 2002).

Opiate tolerance provides a good example to show the association of neuroadaptations of tolerance and withdrawal. The Locus Coeruleus (LC) is expressed important changes in opiate tolerance and withdrawal. Noradrenergic LC efferent fibers project to the prefrontal cortex, VTA, brainstem, and some subcortical areas to excite them. Opioids that bind to µ-opioid receptors on the LC neurons, suppress them and lose the stimulation on its target. In the presence of chronic opiate use, LC neurons compensate for the suppressive effect in case of enhancing the intracellular metabolic machinery to produce noradrenaline.

The sudden abrupt of opiates kindle the enhanced system to initiate the withdrawal symptoms (Trujillo, 2002). At the cellular level, the tolerance of the LC neurons to opiates is coincident with lowering and withdrawal response to increasing the firing rate (Rasmussen, 1991; Rasmussen, Beitner-Johnson, Krystal, Aghajanian, & Nestler, 1990). The ablation of LC from its afferents in vivo or in vitro showed that the withdrawal-induced activation of the LC neurons is dependent on its afferents (Rasmussen & Aghajanian, 1989). It can be postulated that the tolerance and withdrawal at the cellular level are related to the decrease and increase of firing rate.

### 4.2. Tramadol: New analgesic, a new challenge

Tramadol is commonly used in many countries despite its potential for misusing or abusing. The use of tramadol is uncontrolled in some countries. It may have high abuse-risk potential in the long run (Zhang & Liu, 2013), the fact that makes its limitation in the pharmacopeia (Patt & McDiarmid, 2005). Tramadol as an opioid drug may act via multiple mechanisms to change the behavioral responses. The acute and chronic use of tramadol have different effects on the behavioral processes. The mechanism of the tramadol action in analgesia and addiction is not well established (Minami et al., 2015).

Tramadol can easily pass the blood brain barrier to make a central effect on behavior such as depression and motivation (Bertaina-Anglade, Enjuanes, Morillon, & Drieu la Rochelle, 2006; Tetsunaga et al., 2016; Tetsunaga, Tetsunaga, Tanaka, & Ozaki, 2015). The low susceptibility of addiction to tramadol propels its use without any consideration to other perspectives of its side effects. In addition to typical withdrawal symptoms of opiates, the tramadol withdrawal symptoms have other atypical symptoms such as hallucinations, paranoia, extreme anxiety, panic attacks, confusion, and unusual sensory experiences such as numbness and tingling in one or more extremities (Senay et al., 2003).

Unfortunately, the recent reports of the World Health Organization warn about increased misuse and abuse of tramadol in Africa and Western Asia (World Health Organization Expert Committee on Drug Dependence, 2014). Although the addiction to tramadol can interfere with other medications for any clinical trials may important the fluent documentation to choose the right medication in patients with tramadol misuse (Pathak, Kumar, & Rastogi, 2017).

The main challenge in using tramadol is the hidden part of the tramadol function in the body, especially in the brain. There are reports about enhancing the addiction potency of tramadol to initiation to other addictive drugs and vice versa. The opioid-dependent people may be more susceptible to tramadol misuse (Liu et al., 1999; Naslund & Dahlqvist, 2003). The tramadol-induced seizure is a high risk for its long-term use to increase the incidence of serotonin syndrome due to its serotonin reuptake inhibition (McNicol, Midbari, & Eisenberg, 2013).

### 4.3. Tolerance to acute administration of tramadol

In this study, tramadol decreases the firing rate of putative VTA-DA neurons in the early phase of the response. The VTA-DA neurons that had regular firing with low rate showed cellular tolerance as inhibition and cellular dependence as excitation. Tramadol could inhibit the neurons for about 50 min and after that, the excitation lasted about 56 min.

At the cellular and molecular levels, tolerance and dependence are mediated by different signaling mechanisms. The long-term administration of tramadol can produce tolerance due to its opium-like effects that explained previously. The mechanism of chronic-induced tolerance, dependence, and withdrawal to tramadol are probably similar to other opiates. The long-term use of the abused drug could produce global brain changes in the regions that directly mediate addiction. The maladaptation and dysregulated neuronal responses could produce profound somatic or psychological symptoms. Molecular and cellular modifications of the reward system in the chronic administration of the opiates reflect the memory formation for many opiates effects (Rosen, Sun, Rushlow, & Laviolette, 2015).

The acute administration of the opiates is very common in the management of post-operation pain. Acute tolerance is an important issue because of the efficacy of analgesics. Ming et al. showed that the co-administration of the tramadol with dihydroetorphine in rats could produce profound synergistic analgesia, which could delay the onset and development of the tolerance to tramadol (Ming, Wang, Han, & Luo, 2005). The acute tolerance, which is rapidly developed, is related to the interaction of the receptors of the abused drug.

Receptor desensitization and or internalization are perhaps the important response of the neurons to acute exposure to the opiate. The neuroadaptations at different levels can explain the acute tolerance. Receptor tolerance is referred to as the loss of responsiveness of the target receptor over time. The loss of cell surface receptors or functional coupling may produce inhibition of the neuron. In this regard, the involvement of the potassium channels in decreasing the neuronal activity are proposed (Williams, Christie, & Manzoni, 2001).

Recent studies supported the role of intracellular Regulators of receptor Signaling (RGS) molecules. The mice with knockout of RGS9-2 showed an increase in sensitivity and delayed tolerance with exacerbated physical dependence to morphine in acute and chronic administration (Zachariou et al., 2003). The knockout of the RGS9 but not RGS2 in mice, increase potency and duration of the opioid analgesia. The single effective dose of morphine could not show acute tolerance and the development of tolerance was seen after daily intracerebroventricular injection of the opioid for 4 days (Garzon, Rodriguez-Diaz, Lopez-Fando, & Sanchez-Blazquez, 2001).

Actually, tramadol as an opiate analog provides the induction of acute tolerance by modification of the opioid receptors and or other mechanisms. The involvement of opioid receptors with interactions of other neurotransmitter receptors could explain some side effects or bad outcomes of the tramadol in a single dose of administration. The clinical outcomes of the tramadol are better than some opiates and due to its fewer side effects, it is marketed as an Over-The-Counter (OTC) drug in some countries but the precise mechanisms and side effects of the tramadol have remained to be elucidated.

### 4.4. Cellular activity of the VTA-DA neurons to acute administration of tramadol

In vivo, extracellular neuronal activity is a well-known technique to explore the cellular effects of the agents. The firing rate alteration is the most neuronal response that can conduct other studies to evaluate cell signaling. Sevcik et al. reported that tramadol and its main metabolite, O-Desmethyltramadol (O-DT), inhibited the spontaneous discharge of locus coeruleus neurons in prepared slices in a concentration-dependent manner. The effects of (−)-tramadol stopped in the presence of rauwolscine (α2-adrenoceptor antagonist) while the effects of (+)-O-DT virtually disappeared in the presence of naloxone (an opioid antagonist). (+)-Tramadol and (−)-O-DT became inactive only in the presence of naloxone and rauwolscine. (−)-Tramadol and (+)-O-DT can hyperpolarize the membrane potential of LC neurons thus inhibit spontaneous firing.

This effect was abolished by rauwolscine. Naloxone abolishes (+)-O-DT-induced hyperpolarization. The hyperpolarizing effects of noradrenaline can be potentiated in the presence of (−)-tramadol (not in the presence of (+)-O-DT). There is no potentiation of the noradrenaline effect when the cells are hyperpolarized by the current injection to an extent similar to that produced by (−)-tramadol. Both noradrenaline and (−)-tramadol decrease input resistance. These data confirm that the analgesic effects of tramadol are included in both opioid and non-opioid components. It appears that (−)-tramadol inhibits noradrenaline reuptake and via a subsequent increase in the concentration of endogenous noradrenaline, indirectly stimulates α2-adrenoceptors. It seems that (+)-O-DT directly stimulates opioid micro-receptors. The effects of (+)-tramadol and (−)-O-DT consist of combined µ-opioid and α2-adrenergic components (Sevcik, Nieber, Driessen, & Illes, 1993).

Koga et al. (2005) reported that mono-O-dimethyl-tramadol (M1) superfusion could inhibit substantia gelatinosa neurons in vitro in whole-cell patch-clamp recordings. In 41% of neurons, M1 produces outward current at −70 mV that reverses at a potential close to EK. The hyperpolarizing current persists for more than 30 minutes and hardly declines after its washout. This current correlates with DAMGO-induced (a MOR agonist) current in amplitude and is largely reduced by CTAP, an MOR antagonist, not by yohimbine. Noradrenaline produces outward current at −70 mV in a neuron where M1 has no effect on holding currents. M1 can produce persistent hyperpolarization by activating MOR in adult rat SG neurons.

Haeseler et al. (2006) showed that sufentanil, fentanyl, and tramadol reversibly would suppress sodium inward currents at high concentrations when depolarizations started from hyperpolarized holding potential but morphine could not. Short depolarizations inducing fast-inactivation and long induction of pre-pulses slow-inactivation significantly increase blocking potency of tramadol and morphine. Sufentanil, fentanyl, and tramadol block voltage-gated sodium channels.

Tramadol can suppress the amplitude of delayed rectifier K+ current and shift its steady-state inactivation to more negative membrane potential in the NG108-15 neuronal cell line. This effect can stabilize the resting membrane potential and reduce the firing rate of neurons. The effects of tramadol on blocking these channels can explain its antidepressant action (Tsai, Tsai, Wu, & Liu, 2006).

Because of its relation to opioid analgesics, tramadol, whose precise mechanism remains to be elucidated, is commonly used instead of some opioid analgesics because of its low dependence, tolerance, and side effects (Casali, Lepri, Cantini, Landi, & Novelli, 2000; Caspani et al., 2014). While MOR activation and monoamine reuptake inhibition are proposed in the presence of tramadol; there is no evidence for direct action of tramadol on MORs. It may act on ion channels and G Protein-Coupled Receptors (GPCRs) along with monoamine transporters (Minami et al., 2015). It is believed that the antidepressant-like properties of tramadol are related to its monoaminergic reuptake inhibition (Barber, 2011; Caspani et al., 2014; Ferrari, Tiraferri, Palazzoli, & Licata, 2014).

The interaction of tramadol with MORs is well established. Tramadol, morphine, and buprenorphine can produce Conditioned Place Preference (CPP) dose-dependently due to a mechanism that may be mediated by MORs. Tramadol, morphine, and buprenorphine together also produce CPP sub-effectively, but as an adjuvant, potentiate rewarding effects of morphine or buprenorphine (Zhang et al., 2012). Nalbuphine, a қ-opioid, and µ-opioid partial agonist can block tramadol-induced CPP. CPP and antinociception effects of tramadol are related to the dopamine level of VTA-DA neurons (Abdel-Ghany, Nabil, Abdel-Aal, & Barakat, 2015).

As noticed, there is evidence of the interaction between tramadol, MOR, and non-opioid receptors. In heterozygous and homozygous MOR Knockout (MOR-KO) rats, tramadol-induced antinociception reduces significantly. This antinociception is not greatly affected by methysergide, a serotonin receptor antagonist, but is partially blocked by yohimbine, an adrenergic α-2 receptor antagonist, and by both naloxone, a non-selective opioid receptor antagonist, and yohimbine. It is suggested that MOR and the α-2 adrenergic receptor mediate most of the analgesic properties of tramadol (Ide et al., 2006).

Along with these findings, some studies indicate that different opioid and non-opioid agents could change the dopamine level of the brain. The main brain area that is affected by these agents is VTA-DA neurons. These neurons can change the firing rate to affect their targets (Chenu, El Mansari, & Blier, 2009; Rodriguez-Landa, Contreras, Gutierrez-Garcia, & Bernal-Morales, 2003; Werkman et al., 2004; Zhou, Bunney, & Shi, 2006).

In summary, the effect of tramadol on VTA-DA neurons is partly related to the tolerance and dependence of these neurons at the cellular level. Tramadol alone can produce cellular dependence and withdrawal. It may relate to the MORs and other neurotransmissions to produce cellular tolerance and dependence. The results of this study are the first report about the effect of acute tramadol on the VTA-DA neuronal firing for explaining its tolerance and dependence function at the cellular level. Extracellular recording can explore the total neuronal response and this report showed that some VTA-DA neurons could produce the neuronal signaling to tolerance and dependence (withdrawal). The precise evaluation of the putative VTA-DA neurons responses to the acute effects of tramadol needs further cellular and molecular studies to find the key effective factors on these neurons.
